# Identification of two key biomarkers *CD93* and *FGL2* associated with survival of acute myeloid leukaemia by weighted gene co‐expression network analysis

**DOI:** 10.1111/jcmm.18552

**Published:** 2024-07-25

**Authors:** Haijun Han, Jie Liu, Shengyu Zhu, Tiejun Zhao

**Affiliations:** ^1^ Key Laboratory of Novel Targets and Drug Study for Neural Repair of Zhejiang Province School of Medicine, Hangzhou City University Hangzhou China; ^2^ College of Life Sciences, Zhejiang Normal University Jinhua China

**Keywords:** AML, *CD93*, *FGL2*, survival, WGCNA

## Abstract

Acute myeloid leukaemia (AML) is a biologically heterogeneous haematological malignancy. This study was performed to identify the potential biomarkers for the prognosis and treatment of AML. We applied weighted gene co‐expression network analysis to identify key modules and hub genes related to the prognosis of AML using data from The Cancer Genome Atlas (TCGA). In total, 1581 differentially expressed genes (1096 upregulated and 485 downregulated) were identified between AML patients and healthy controls, with the blue module being the most significant among 14 modules associated with AML morphology. Through functional enrichment analysis, we identified 217 genes in the blue module significantly enriched in ‘neutrophil degranulation’ and ‘neutrophil activation involved in immune response’ pathways. The survival analysis revealed six genes (*S100A9*, *S100A8*, *HK3*, *CD93*, *CXCR2* and *FGL2*) located in the significantly enriched pathway that were notably related to AML survival. We validated the expression of these six genes at gene and single‐cell levels and identified methylation loci of each gene, except for *S100A8*. Finally, in vitro experiments were performed to demonstrate whether the identified hub genes were associated with AML survival. After knockdown of *CD93* and *FGL2*, cell proliferation was significantly reduced in U937 cell line over 5 days. In summary, we identified *CD93* and *FGL2* as key hub genes related to AML survival, with *FGL2* being a novel biomarker for the prognosis and treatment of AML.

## 1 INTRODUCTION

Acute myeloid leukaemia (AML) is a haematological malignancy characterized by arrested differentiation and abnormal proliferation of immature bone marrow cells, and it is the most common acute leukaemia in adults.^1^ The age‐adjusted incidence of AML is 4.3 per 100,000, and the mortality rate is approximately 3 per 100,000 in the United States, which are higher than the other three subtypes of leukaemia.^2^ The 5‐year survival rate of AML is <30%, indicating a poor prognosis.^2^, ^3^ Relapse is common in patients undergoing AML treatment, occurring in 10%–40% of younger patients and as much as 40%–60% in patients aged >60 years.^4^ Although progress have been made in the effectiveness of intensive chemotherapy and targeted therapies,^5^, ^6^ more specific molecular targets for the prognosis and treatment of AML are needed.

Increasing numbers of potential biomarkers associated with AML prognosis and therapy have been identified.^7^, ^8^ For example, mutations in *FLT3* (fms‐related receptor tyrosine kinase 3), *IDH1/2* (isocitrate dehydrogenase (NADP(+)) 1/2), *NPM1* (nucleophosmin 1), *CD33*, *TP53* (tumour protein p53) and *ASXL1* (ASXL transcriptional regulator 1) have become targeted therapy biomarkers in clinic.^7^ High expression levels of *IFITM3* (interferon‐induced transmembrane protein 3),^9^ *NAT10* (N‐acetyltransferase 10),^10^ *TET3*,^11^ *PPM1D* (protein phosphatase Mg^2+^/Mn^2+^‐dependent 1D)^12^ and *Wnt11* ^13^ have been identified as poor prognostic biomarkers in patients with AML, potentially guiding AML management. Downregulation of *SH3BGRL* (SH3‐domain‐binding glutamic acid‐rich protein‐like protein) is associated with poor outcomes of AML. Knockdown of *SH3BGRL* in MV4, ML‐1 and MOLM‐13 AML cell lines notably promotes cell proliferation and cell cycle progression, indicating the potential use of *SH3BGRL* as a diagnostic and prognostic marker in AML.^14^ Similarly, both in vitro and in vivo experiments have shown that knockdown of Acyl‐CoA synthetase long‐chain family member 5 suppresses cell proliferation and increases cell apoptosis, indicating that it may serve as a potential prognostic marker for AML.^15^ However, the complexities in AML go beyond single gene aberrations. More importantly, targeted treatments are only effective for particular subsets of patients with AML, and many patients are not targetable.^6^ Thus, there is a clear need to identify additional biomarkers.

With the rapid development of genomic technologies, numerous datasets have been generated by next‐generation sequencing and made publicly available.^16^ This provides the opportunity to identify targets related to the diagnosis and prognosis of disease. The weighted gene co‐expression network analysis (WGCNA) is a compelling method that utilizes big data to detect synergistically expressed genes related to clinical phenotypes rather than focusing on a single gene.^17^ WGCNA has recently become widely used in various diseases, including AML, to identify target hub genes.^18^, ^19^, ^20^, ^21^ However, there is almost no overlap among these genes, and their mechanisms of action are largely unknown. Therefore, it is necessary to obtain more reliable targets related to AML by integrating multi‐omics data.

The present study was performed to identify novel biomarkers associated with AML survival. First, we identified differentially expressed genes (DEGs) between healthy controls and patients with AML using data from The Cancer Genome Atlas (TCGA) database. These genes were then applied to WGCNA. Second, enrichment analysis was performed for genes in the key module identified as being related to AML. Third, survival‐associated hub genes were identified by performing survival analyses for genes in the top two enriched pathways. Fourth, the expression of hub genes was validated at different levels, and the methylation loci of these genes were identified. Finally, we knocked down the candidate hub genes and demonstrated that two of these genes were key hub genes related to survival of patients with AML.

## 2 MATERIALS AND METHODS

### 2.1 Data collection and processing

The AML data, which comprised 150 samples (100 AML samples and 50 healthy control samples), were obtained from TCGA data portal (https://portal.gdc.cancer.gov/).^16^ According to the French‐American‐British (FAB) classification, AML was classified into eight subtypes: undifferentiated AML (M0), AML with minimal maturation (M1), AML with maturation (M2), acute promyelocytic leukaemia (M3), acute myelomonocytic leukaemia (M4), acute monocytic leukaemia (M5), acute erythroid leukaemia (M6) and acute megakaryoblastic leukaemia (M7). The dataset contains mRNA expression data derived from the HiSeq RNA Sequencing platform (Illumina, San Diego, CA, USA) and clinical data, including leukaemia morphology and patient age, gender and survival time.

### 2.2 Identification of DEGs

DEGs between the M0–M1 and M2–M7 subtypes were screened using the R package ‘DESeq2’.^22^ An adjusted *p* value <0.05 and |Log_2_ FC| ≥1 were used as cut‐offs to identify significant DEGs, which were presented in a volcano plot using the R package ‘ggplot2’.

### 2.3 WGCNA

The R package ‘WGCNA’ was used to construct a co‐expression network of the DEGs.^17^ Briefly, ‘hclust’ was used to evaluate the expression matrix of each sample. The scale independence and mean connectivity were calculated. The adjacency matrix was then transformed into a topological overlap matrix. Hierarchical clustering was performed to identify significant module eigengenes. The soft‐threshold parameter was set to five, each module included at least 30 genes, and other parameters were used at their default values. The correlation between each module and clinical traits was determined.

### 2.4 Functional enrichment analysis and protein–protein interaction network construction

To determine the specific biological functions of genes in the key module, we performed gene ontology (GO) enrichment analysis using the ‘clusterProfiler’ R package.^23^ An adjusted *p* value <0.05 was considered statistically significant for the enrichment analysis. Genes in the top two GO terms were then used to construct a protein–protein interaction (PPI) network using the online database STRING (https://string‐db.org/). The minimum required interaction score was set to 0.4.

### 2.5 Survival analysis of genes in key module

To screen candidate hub genes, all genes in the key module were subjected to survival analysis using AML data from TCGA in accordance with previously published methods.^24^, ^25^ Briefly, patients with AML were grouped into high‐expression and low‐expression subgroups based on the median expression values. A Kaplan–Meier curve for overall survival was presented for each gene using the R package ‘survival’.^26^ The follow‐up cut‐off was set at the median quartile. A receiver operating characteristic (ROC) curve analysis was conducted using the R package ‘pROC’ to further validate the prognostic importance of the biomarkers. Genes with a *p* value <0.05 for overall survival rate were defined as the candidate hub genes.

### 2.6 Validation of expression levels of candidate hub genes

To validate the reliability of the identified candidate hub genes, we applied the online database GEPIA (http://gepia.cancer‐pku.cn/index.html) to determine the expression of each gene in patients with AML, and the expression levels were presented as boxplots.^27^ Briefly, each candidate hub gene symbol or gene ID was entered into the ‘Gene’ field, with the cut‐offs for |Log_2_ FC| and *p* value set at 1 and 0.01, respectively. The dataset was selected as ‘LAML’, and the boxplot of each gene was obtained. Furthermore, we used the UALCAN (http://ualcan.path.uab.edu) database to evaluate the expression of the above genes in each subtype of AML.^28^ Each candidate hub gene symbol was entered into the ‘Scan by gene(s)’ field. After selecting the AML dataset, we obtained the expression of each gene in AML based on the FAB classification. Statistical analysis was performed using student's *t*‐test compared to M0, with a *p* value <0.05 considered statistically significant.

### 2.7 Expression of candidate hub genes at single‐cell level

Single‐cell RNA sequencing technology provides a comprehensive understanding of gene function at the single‐cell level. The Tumour Immune Single‐cell Hub (TISCH) (http://tisch.comp‐genomics.org/home/) is a database integrating extensive single‐cell transcriptomic profiles across 28 cancer types.^29^ In the present study, the AML_GSE116256 dataset was used because it includes 16 patients with AML and 5 healthy donors profiling 38,410 cells from 40 bone marrow aspirates. This is the largest dataset in TISCH available for selection.^30^ Data were presented and compared across cell types (major lineages) and grouped by source of samples (bone marrow from healthy donors or patients with AML). Statistical analysis was conducted using the Mann–Whitney *U* test, with a *q* value <0.05 considered significant.

### 2.8 Methylation analysis of candidate hub genes

To further understand the role of candidate hub genes in AML, we perform a methylation analysis using MEXPRESS (http://mexpress.be), a web tool that integrates and visualizes expression, DNA methylation and clinical TCGA data at the single‐gene level.^31^ Briefly, the gene name was entered into the text field, and after selecting AML as the cancer type, we plotted the expression and methylation of the candidate hub gene in AML. The methylation loci in the promoter region of each gene were identified. The correlation between gene expression and methylation levels was also obtained and expressed with Pearson correlation coefficients. All *p* values were adjusted using the Benjamini–Hochberg method.

### 2.9 Cells

Peripheral blood mononuclear cells (PBMCs) were collected from healthy donors at Zhejiang Normal University Hospital. A 10 mL sample of whole blood from a healthy donor was layered on 5 mL of Ficoll‐Paque medium in a 15 mL centrifuge tube, then centrifuged at 1800 rpm for 30 min at room temperature without braking. PBMCs were recovered from the Ficoll–Paque medium and washed three times with PBS. Clinical samples were obtained and used according to the principles of the Declaration of Helsinki. The experiment was approved by the Ethics Committee of Zhejiang Normal University (approval no. ZSRT2023007). Four AML cell lines (U937, THP‐1, OCI‐AML3 and MOLM‐13) and the PBMCs were cultured in RPMI 1640 supplemented with 10% fetal bovine serum (FBS) and antibiotics.^32^ The 293FT cell line was cultured in Dulbecco's modified Eagle medium supplemented with 10% FBS and antibiotics.

### 2.10 Knockdown of target genes

Knockdown of *CD93* and *FGL2* in AML cell line U937 was performed with a lentiviral vector pLKO.1‐based short hairpin RNA (shRNA) system (Open Biosystems, Huntsville, AL, USA) as previously described.^32^ Briefly, shRNA sequences targeting *CD93* and *FGL2* were cloned into the pLKO.1 vector. 293FT cells were transfected with 15 μg of pLKO.1 vector, 15 μg of pCMV‐△8/9 and 7.5 μg of pcDNA‐VSV‐G using Lipofectamine 2000 (Thermo Fisher Scientific, Waltham, MA, USA). Forty‐eight hours later, the supernatant was collected, and the virus was concentrated at 25,000 rpm for 2 h at 4°C. U937 cells were transduced with viral supernatant and 4 μg/mL polybrene. Forty‐eight hours after infection, stable transfectants were selected using G418 (600 μg/mL). The shRNA sequences specifically targeting *CD93* and *FGL2* were: shCD93, 5′‐GCCTTACTCTAACTGGCACAA‐3′; and shFGL2, 5′‐GCATTACGTTTCAACAAACAT‐3′.

### 2.11 Real‐time quantitative polymerase chain reaction

Total RNA was isolated using TRIzol Reagent (Thermo Fisher Scientific) as previously described.^32^ We reverse‐transcribed 1 μg of total RNA into single‐stranded cDNA using random primers and the SuperScript III reverse transcriptase (Life Technologies, Carlsbad, CA, USA). RT‐qPCR was performed using Power SYBR Green PCR Master Mix and StepOnePlus Real‐Time PCR System (Thermo Fisher Scientific). Each 10 μL reaction volume contained 4 μL cDNA, 5 μL 1 × SYBR Green SuperReal Premix (Tiangen, Beijing, China), 0.2 μL ddH_2_O and 0.4 μL each primer. The reaction conditions included an initial denaturation step at 95°C for 15 min, followed by 40 cycles at 95°C for 10 s, 60°C for 32 s and 72°C for 30 s with fluorescent signal recording. The normalized value in each sample was derived from the relative quantity of target mRNA divided by the relative quantity of 18S rRNA. The primers for *CD93*, *FGL2* and 18S rRNA were as follows: *CD93* forward, 5′‐TGGAGAACCAGTACAGTCCGA‐3′ and reverse, 5′‐TCCAAGGGGCCTTTAAGGAG‐3′; *FGL2* forward, 5′‐ACTGTGACATGGAGACCATG‐3′ and reverse, 5′‐TCCTTACTCTTGGTCAGAAG‐3′; and 18S forward, 5′‐AACCCGTTGAACCCCATT‐3′ and reverse, 5′‐CCATCCAATCGGTAGTAGCG‐3′.

### 2.12 Cell proliferation assay

Cells were seeded into 96‐well plates at the indicated time points. A 3‐(4,5‐dimethythiazol‐2‐yl)‐2,5‐diphenyltetrazolium bromide (MTT) assay was performed to assess cell proliferation as previously described.^32^ Briefly, U937 cells were seeded at a density of 4 × 10^4^ cells per well in 96‐well plates, and 10 μL of MTT solution was added at the indicated time points (1–5 days). The cells were incubated at 37°C for 2 h and then lysed with 100 μL of lysis buffer (4% Triton X‐100 and 0.14% HCl in 2‐propanol). The absorbance at 595 nm was measured using a microplate reader (Bio‐Rad, Hercules, CA, USA).

### 2.13 Statistical analysis

For the in vitro experiments, all values are presented as mean ± standard error of the mean, and differences were determined using the unpaired *t*‐test with GraphPad Prism v.8.0 software (GraphPad Software, San Diego, CA, USA). A *p* value <0.05 was considered statistically significant.

## 3 RESULTS

### 3.1 Screening of DEGs between healthy controls and patients with AML

**FIGURE 1:**
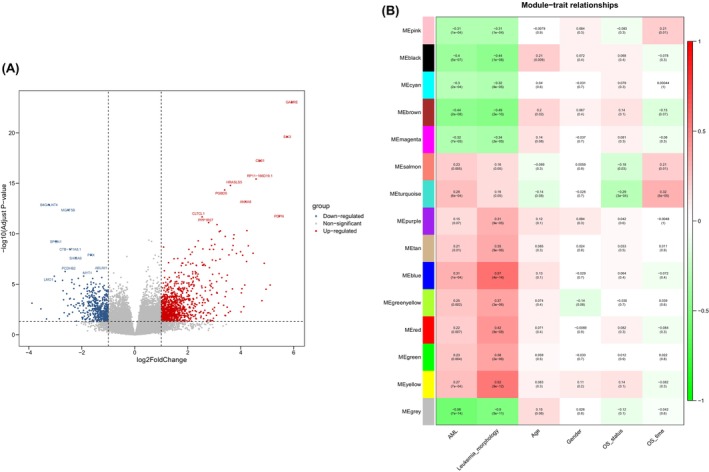
(A) Volcano plot of differentially expressed genes (DEGs). The cut‐off values are *p*_adj_ <0.05 and |Log_2_ FC| ≥1. Red and green dots indicate upregulated and downregulated genes, respectively. Grey dots indicate no differences in RNA expression. (B) Heatmap of the correlation between each module and clinical traits of AML.

As shown in Table S1, this study included 50 healthy controls and 100 patients with AML. The median age of the healthy controls and patients with AML was 54.82 and 53.65 years, respectively. By comparing the gene expression in each group, we obtained 1581 DEGs (*p*_adj_ <0.05 and |Log_2_ FC| ≥1), with 1096 genes upregulated and 485 genes downregulated (Figure 1A).

### 3.2 Identification of key module and hub genes related to AML by WGCNA

To identify the key module and hub genes related to AML, we performed WGCNA using the above‐mentioned DEGs. To ensure a scale‐free network, we selected a power of five and a scale‐free *R* ^2^ = 0.76 as the soft‐thresholding parameters (Figure S1A,B). We then compared the correlations of the DEGs, clustering the co‐expressed genes into modules. All generated modules showed strong associations with each other (Figure S1C,D). Finally, we identified 14 modules associated with AML morphology (Figure 1B). Of these, the blue module was the most significant (*R* ^2^ = 0.57, *p* = 4 × 10^−14^) and was used for further analysis.

**FIGURE 2:**
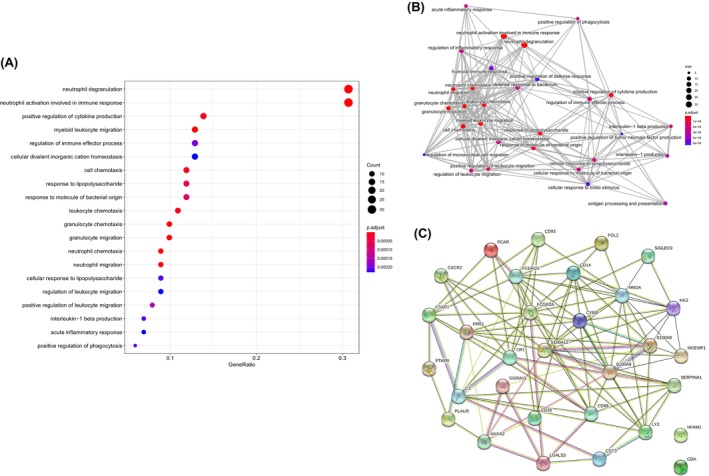
Functional enrichment analysis. (A) Gene Ontology (GO) enrichment analysis for the genes in the blue module. (B) Network of enriched GO clusters. (C) The protein–protein interaction (PPI) network analysis of the genes in top two GO terms.

The blue module included 217 genes. As shown in Figure 2A, these genes were significantly enriched in ‘neutrophil degranulation’ and ‘neutrophil activation involved in immune response’ pathways (*p*_adj_ = 5.1 × 10^−22^). Figure 2B shows the network of the markedly enriched GO terms. These two GO terms included 31 genes/proteins, which also showed significant protein–protein interactions (*p* = 1 × 10^−16^) (Figure 2C). These proteins were used for further survival analysis.

### 3.3 Validation of expression of survival‐related genes in AML

**FIGURE 3:**
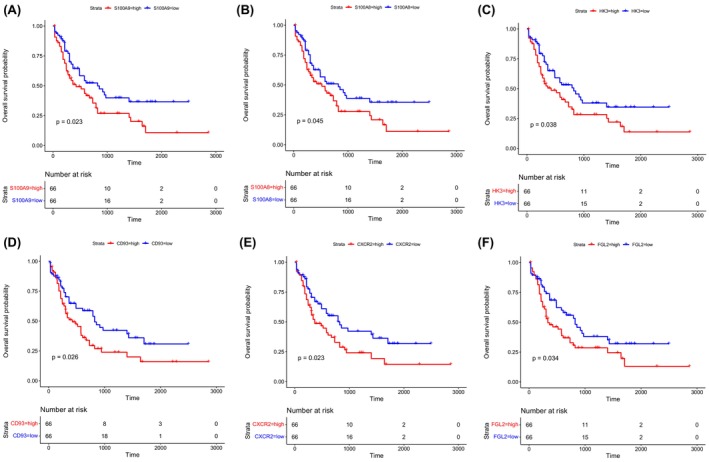
Kaplan–Meier survival analysis of candidate hub genes in AML. (A–F) Survival analysis of *S100A9*, *S100A8*, *HK3*, *CD93*, *CXCR2* and *FGL2* in the blue module. Data are based on the AML samples from The Cancer Genome Atlas database.

To explore the survival‐related genes in AML, we performed a Kaplan–Meier survival analysis of the 31 genes mentioned above. After screening, we identified six genes (*S100A9*, *S100A8*, *HK3*, *CD93*, *CXCR2* and *FGL2*) that were significantly related to the overall survival probability of AML (*p* < 0.05) (Figure 3A–F). Patients with AML who showed high expression of these genes had shorter survival. Furthermore, we performed ROC analysis of these six identified genes. As shown in Figure S2, the AUC of these genes was close to or >0.7, indicating good sensitivity and specificity in predicting overall survival in patients with AML. Thus, these six genes were selected as the candidate hub genes related to survival of patients with AML.

**FIGURE 4:**
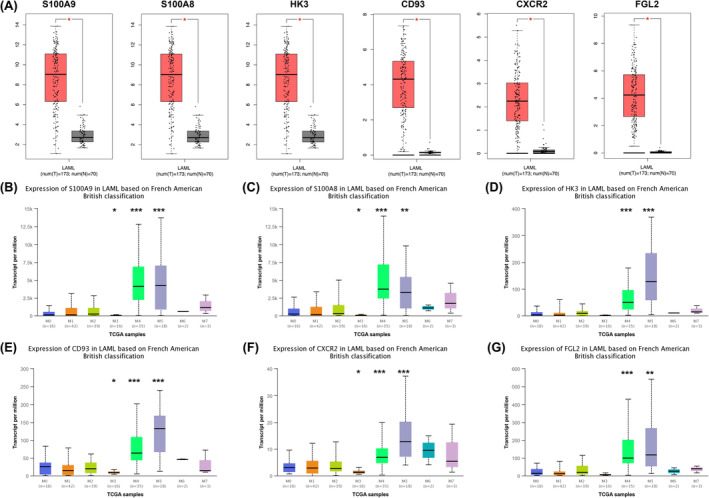
Expression of six candidate hub genes in AML. (A) Expression of *S100A9*, *S100A8*, *HK3*, *CD93*, *CXCR2* and *FGL2* in AML (red box) and healthy controls (grey box). (B–G) Expression of *S100A9*, *S100A8*, *HK3*, *CD93*, *CXCR2* and *FGL2* in eight subtypes of AML based on FAB classification. *p < 0.05, **p < 0.01, ***p < 0.001.

Next, we examined the expression of these hub genes in different subtypes and cell types of AML. As shown in Figure 4A, these six genes were significantly upregulated in patients with AML (*p* < 0.05), consistent with the results in Figure 1A. According to the FAB classification, these genes presented varying expression in each subtype (Figure 4B–G). Specifically, *S100A9*, *S100A8*, *CD93* and *CXCR2* were significant in the M3 (*p* < 0.05), M4 (*p* < 0.001) and M5 (*p* < 0.001) subtypes, while *HK3* and *FGL2* only showed significance in the M4 (*p* < 0.001) and M5 (*p* < 0.001) subtypes. These findings indicate that these genes mainly play critical roles in the M3, M4 and M5 subtypes of AML.

**FIGURE 5:**
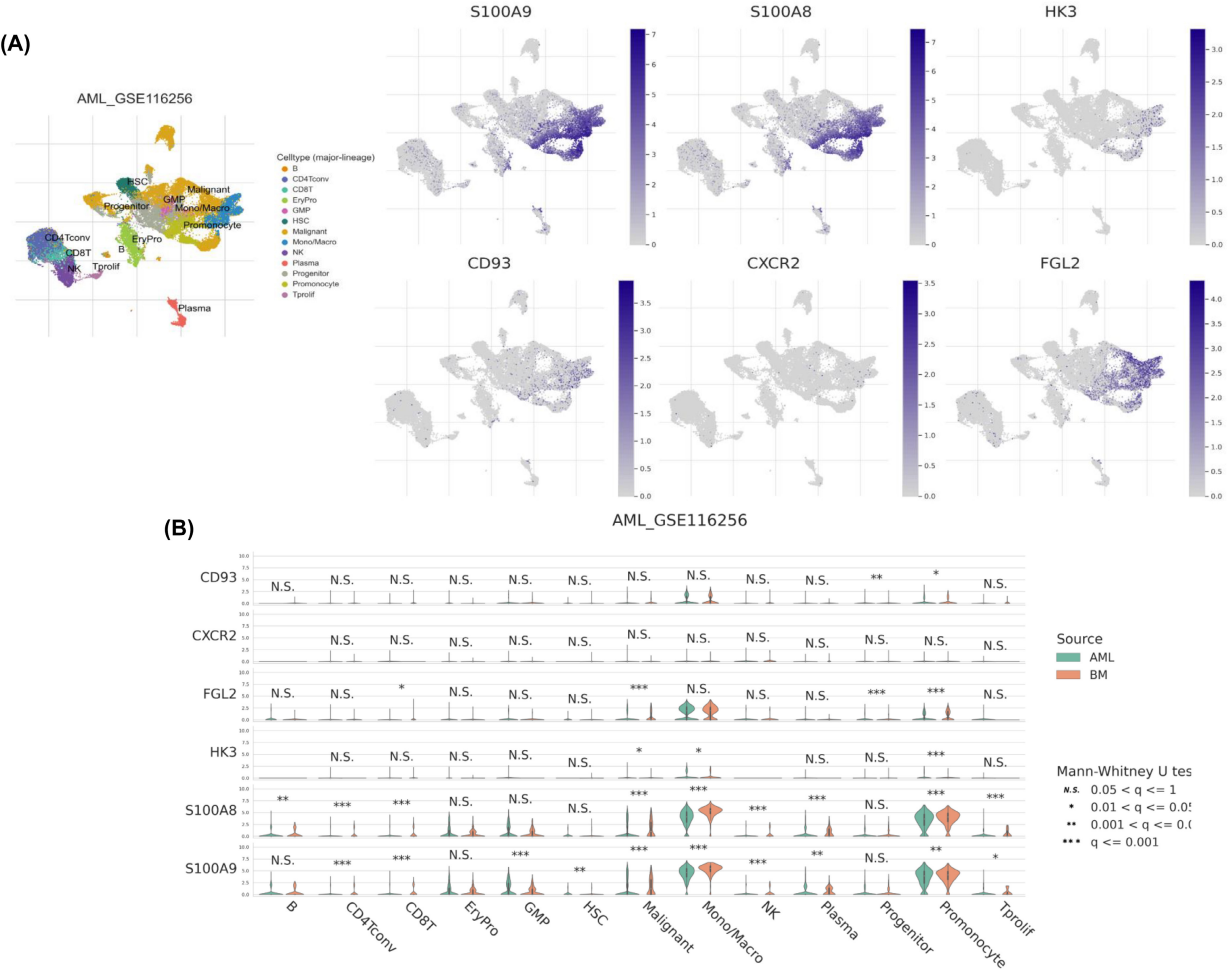
Expression of six candidate hub genes at the single‐cell level in AML. (A) Overview of *S100A9*, *S100A8*, *HK3*, *CD93*, *CXCR2* and *FGL2* gene expression among all cell types. (B) Expression of *S100A9*, *S100A8*, *HK3*, *CD93*, *CXCR2* and *FGL2* across different cell types and grouped by source. The presented data were compared between patients with AML (green) and BM donors (red). BM, bone marrow.

Furthermore, we determined the expression of these six hub genes at the single‐cell level (Figure 5). The results indicated that *S100A9* and *S100A8* were highly expressed in monocytes/macrophages and promonocyte cells, showing statistical significance in most cell types (*q* < 0.05), except for *S100A9* in B cells, erythroblasts and progenitor cells and *S100A8* in erythroblasts, granulocyte–monocyte progenitors, haematopoietic stem cells and progenitor cells (*q* > 0.05). Among the other four candidate hub genes, *HK3* showed significance only in malignant cells, monocytes/macrophages and promonocyte cells (*q* < 0.05); *FGL2* only in CD8^+^ T cells, malignant cells, progenitor cells and promonocyte cells (*q* < 0.05); and *CD93* only in progenitor and promonocyte cells (*q* < 0.05). However, *CXCR2* did not show significance in any cell types between healthy control bone marrow and patients with AML (*q* > 0.05).

### 3.4 Identification of methylation loci of candidate hub genes in AML

**FIGURE 6:**
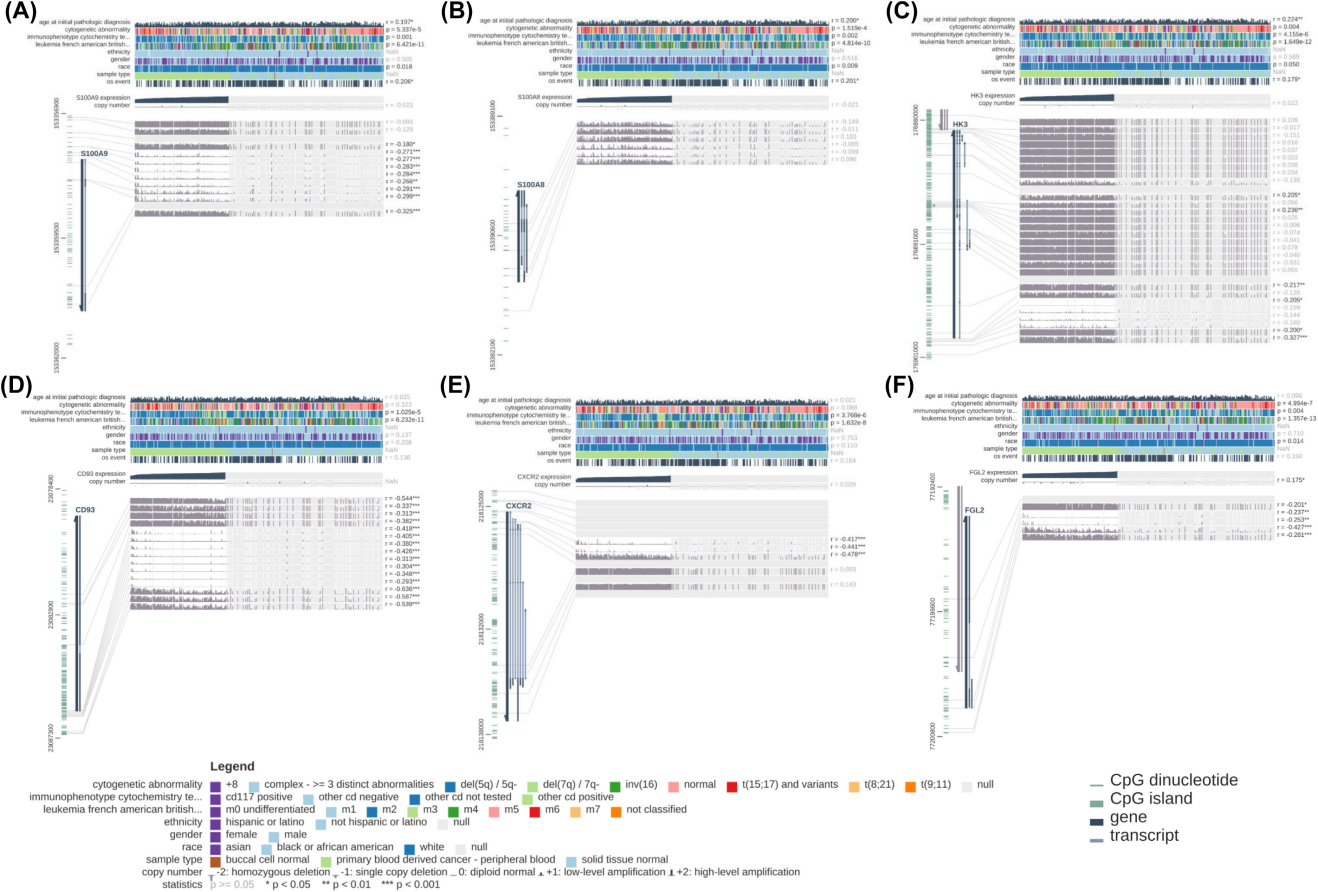
(A–F) Methylation analysis of *S100A9*, *S100A8*, *HK3*, *CD93*, *CXCR2* and *FGL2* genes in AML around CpG islands and promoter regions.

To further understand the role of the hub genes in AML, we analysed the methylation levels of these genes using the MEXPRESS tool. The results (Figure 6) showed 9, 6, 15, 3 and 5 significant methylation loci in *S100A9*, *HK3*, *CD93*, *CXCR2* and *FGL2* (*p* < 0.05), respectively, except for *S100A8* (*p* > 0.05) (Figure 6B). These loci were significantly negatively correlated with the expression of their respective genes (*p* < 0.05) (Table S2). It suggests that these methylation loci may play important roles in regulating the candidate hub genes in AML.

### 3.5 Relationship of *CD93* and *FGL2* with survival in AML

**FIGURE 7:**
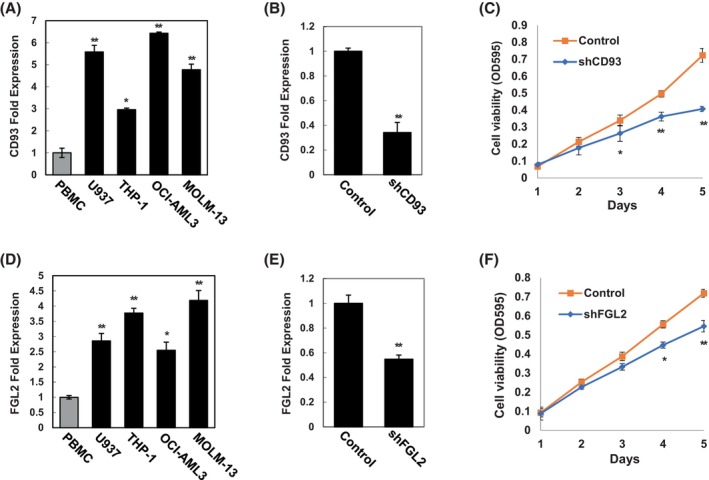
Validation of *CD93* and *FGL2* related to survival of AML. (A, D) Expression of *CD93* and *FGL2* in various AML cell lines including U937, THP‐1, OCI‐AML3 and MOLM‐13. (B, E) Knockdown of *CD93* and *FGL2* in U937 cell line. (C, F) Cell viability determination by MTT assay after *CD93* and *FGL2* knockdown. *p < 0.05, **p < 0.01.

Finally, to demonstrate the role of candidate hub genes related to survival in AML, we measured the expression of these genes in various leukaemia cell lines and explored their involvement in survival by knockdown experiments. The results showed that *CD93* and *FGL2* were differentially expressed in the U937, THP‐1, OCI‐AML3 and MOLM‐13 cell lines (Figure 7A,D). To clarify the role of *CD93* and *FGL2* in AML cells, we knocked down *CD93* and *FGL2* using shRNA in the U937 cell line. As shown in Figure 7B,E, *CD93* and *FGL2* were successfully knocked down in U937 cells (*p* < 0.01). Furthermore, the MTT assay demonstrated that knockdown of *CD93* attenuated U937 cell viability on Day 3 (*p* < 0.05), Day 4 (*p* < 0.01) and Day 5 (*p* < 0.01), while knockdown of *FGL2* reduced cell viability only on Day 4 (*p* < 0.05) and Day 5 (*p* < 0.01). These data demonstrated that *CD93* and *FGL2* are involved in the survival of AML cells, and these two genes were defined as key hub genes related to survival in AML.

## 4 DISCUSSION

In this study, we identified 1581 DEGs by comparing gene expression between patients with AML and healthy controls. By performing WGCNA, we obtained 14 modules associated with the phenotype of AML, with the blue module consisting of 217 genes being the most significant. These genes were significantly enriched in the ‘neutrophil degranulation’ and ‘neutrophil activation involved in immune response’ pathways. Among the genes in these pathways, six genes (*S100A9*, *S100A8*, *HK3*, *CD93*, *CXCR2* and *FGL2*) were significantly related to the survival of patients with AML. These genes mainly play important roles in the M4 and M5 subtypes of AML based on the FAB classification and in CD8^+^ T cells, malignant, monocytes/macrophages, promonocytes and progenitor cells. Finally, through in vitro experiments, we demonstrated that *CD93* and *FGL2* are related to cell survival and can be used as prognostic biomarkers for AML.

Co‐expression network analysis has been widely used to discover key modules and hub genes as candidate biomarkers related to diseases, especially in cancer studies.^17^ However, few such studies have focused on AML. Yu et al.^33^ recently used WGCNA to show that high expression of *LOC541471*, *GDAP1*, *SOD1* and *STK25* is associated with poor survival of patients with AML. Xu et al.^34^ also used WGCNA to show that *TRIM32* is associated with a poor prognosis in patients with AML. Lai et al.^25^ identified *FNDC3B*, *VSTM1*, *PLA2G4A*, *GOLGA3* and *CALR* as key prognostic biomarkers that may guide the treatment of patients with AML. Xie et al.^35^ identified a group of immune‐related genes (including *CTSD*, *GNB2*, *CDK6* and *WAS*) associated with serum interleukin (IL)‐33 expression in the prognosis of AML. Zhu et al.^18^ found that the hub gene *CEACAM5* is significantly associated with the prognosis of AML and can serve as a potential target for AML treatment. Wang et al.^20^ identified a group of hub genes (*NFE2*, *TRIM27*, *MEF2C*, *ETS1*, *TAL1*, *FOXO1* and *GATA1*) and pathways in AML using WGCNA. In the present study, we identified six hub genes related to survival of patients with AML by applying WGCNA. We validated two genes (*CD93* and *FGL2*) as the real hub genes that can serve as prognostic biomarkers of AML through in vitro studies.

Of these hub genes, *S100A9*, *S100A8*, *HK3*, *CD93* and *CXCR2* have been previously reported. However, *FGL2* is a novel target that may also play a significant role in AML. *S100A8* and *S100A9* are members of the S100 protein family and are involved in various cellular processes, including proliferation, differentiation and migration. Overexpression of *S100A8* and *S100A9* is related to a poor prognosis in patients with AML. These genes are regulated through the TLR4 or IL‐6/Jak/STAT3 signalling pathway, and have the potential for use in AML therapy.^36^, ^37^, ^38^ *HK3* codes for a glycolytic enzyme that serves as the primary hexokinase member most frequently expressed in myeloid cells. The expression of this gene has been reported to be significantly decreased in patients with AML, playing a role in neutrophil differentiation and myeloid cell survival.^39^, ^40^, ^41^ *CXCR2* code for a seven‐transmembrane domain G‐protein‐coupled receptor (the receptor of IL‐8). This gene is highly expressed in multiple types of leukaemic cells. It is overexpressed in patients with AML and is considered a poor prognostic factor and a potential therapeutic target in AML.^42^, ^43^ *CD93*, which codes for a transmembrane receptor belonging to the C‐type lectin family, is widely expressed in various cells including myeloid cells, platelets, early B‐cell precursors and endothelial cells.^44^ It is also involved in the process of cell proliferation, cell migration and tumour angiogenesis and is considered a therapeutic target of AML.^45^, ^46^ Consistent with these findings, we also identified these genes as being associated with the survival of patients with AML.

Interestingly, we found that the novel gene *FGL2* is also related to AML survival. *FGL2* protein belongs to the fibrinogen‐associated protein family, which plays significant roles in tumour development and the immune microenvironment of tumours.^47^ Both *CD93* and *FGL2* are membrane‐bound proteins, making them highly useful as potential targets for chimeric antigen receptor‐modified T (CAR‐T) cell therapy, especially in haematological malignancies.^48^ *CD93* and *FGL2* were recently highlighted as promising CAR‐T cell targets in AML or other cancers.^49^, ^50^ Although *FGL2* has been identified as a biomarker in various cancers, such as glioma,^51^, ^52^, ^53^ breast cancer,^54^ gastrointestinal stromal tumour^55^ and lung cancer,^56^, ^57^ no studies have focused on *FGL2* as a prognostic and treatment biomarker in AML. Here, we demonstrated that *FGL2* is associated with the survival and viability of AML patients and cells through comprehensive analyses, indicating its potential as a biomarker and CAR‐T cell target for AML diagnosis and therapy. Considering the vast heterogeneity within AML, a larger dataset or additional clinical samples are needed to validate the above findings. Additionally, given the vast heterogeneity in AML, approaches such as dimensionality reduction and overlaying the identified biomarkers with canonical signature genes are necessary to yield more definitive insights into the heterogeneity underlying AML.^58^

## 5 CONCLUSIONS

We identified two key hub genes (*CD93* and *FGL2*) related to AML survival through WGCNA and in vitro knockdown experiments. Of these two genes, *FGL2* is a novel potential biomarker for the prognosis and treatment of AML. However, the detailed mechanism and critical roles of *FGL2* in AML progression require further investigation.

## AUTHOR CONTRIBUTIONS

**Haijun Han:** Conceptualization (lead); data curation (lead); funding acquisition (lead); investigation (lead); project administration (lead); writing – original draft (lead); writing – review and editing (lead). **Jie Liu:** Data curation (equal); validation (lead). **Shengyu Zhu:** Data curation (equal); validation (lead). **Tiejun Zhao:** Conceptualization (supporting); funding acquisition (lead); investigation (supporting); project administration (supporting); supervision (supporting); validation (supporting); writing – review and editing (supporting).

## FUNDING INFORMATION

This work was supported by grants from the National Natural Science Foundation of China to T.Z. (Nos. 32370147 and 31970173), a grant from the Natural Science Foundation of Zhejiang Province to H.H. (No. LQ24C090002), a grant from the Special Support Program for High‐level Talents in Zhejiang Province to T.Z. (No. 2023R5242), and a grant from Hangzhou Science and Technology Bureau to H.H.

## CONFLICT OF INTEREST STATEMENT

There are no financial interests or potential conflicts of interest.

## Supporting information


Figure S1.
Figure S2.

Table S1.

Table S2.

## Data Availability

The raw sequencing data supporting the conclusions of this paper were obtained from TCGA data portal (https://portal.gdc.cancer.gov/). All data generated or analysed during this study are included in this published article and its supplementary information files.
