# Supplementary figures and images for: Identification of two key biomarkers CD93 and FGL2 associated with survival of acute myeloid leukaemia by weighted gene co‐expression network analysis
Source: J Cell Mol Med. 2024 Jul 25;28(14):e18552. doi: 10.1111/jcmm.18552 (PMC11272607; doi:10.1111/jcmm.18552)

## Slide 1
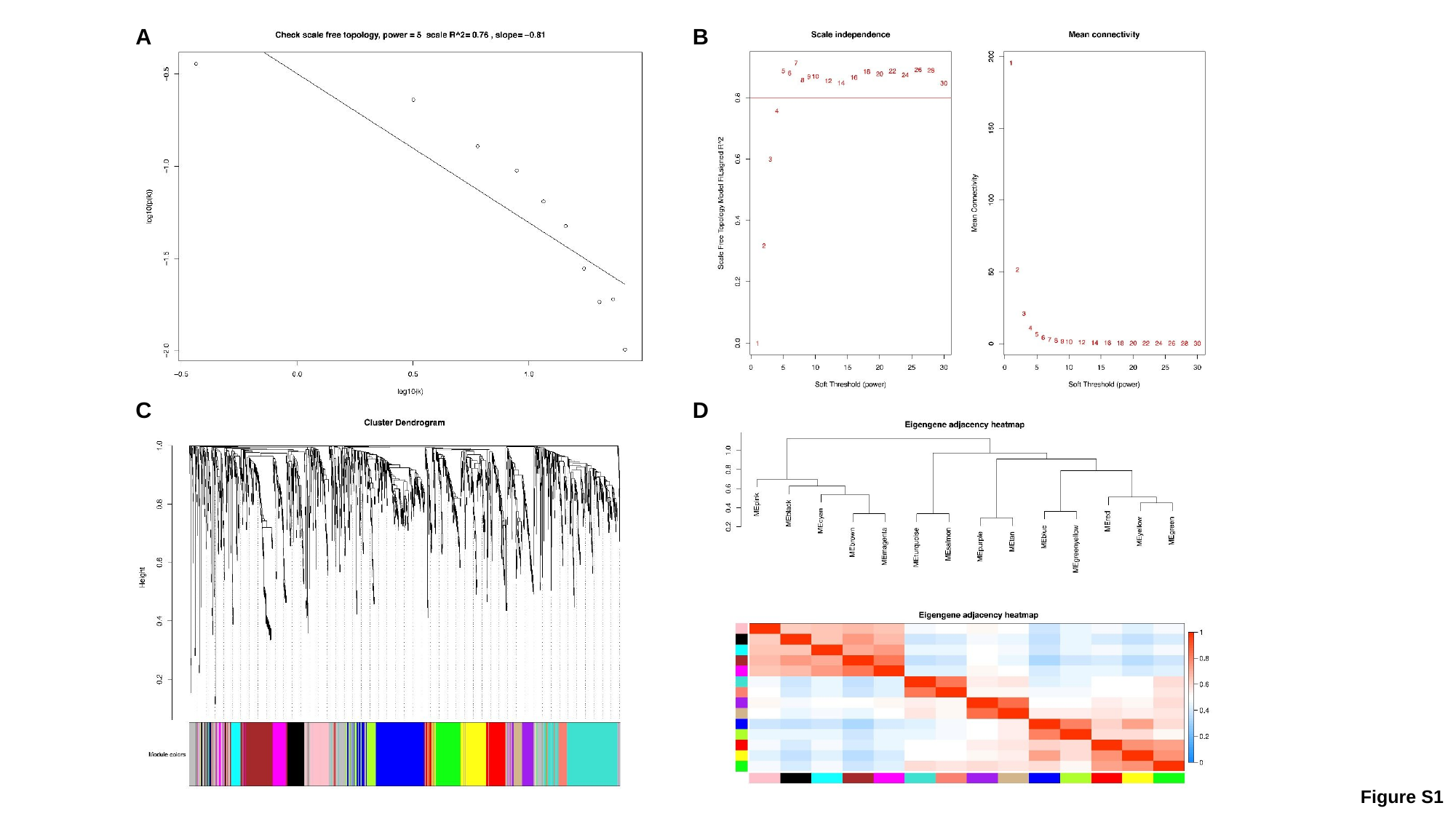

A
B
C
D
Figure S1

## Slide 2
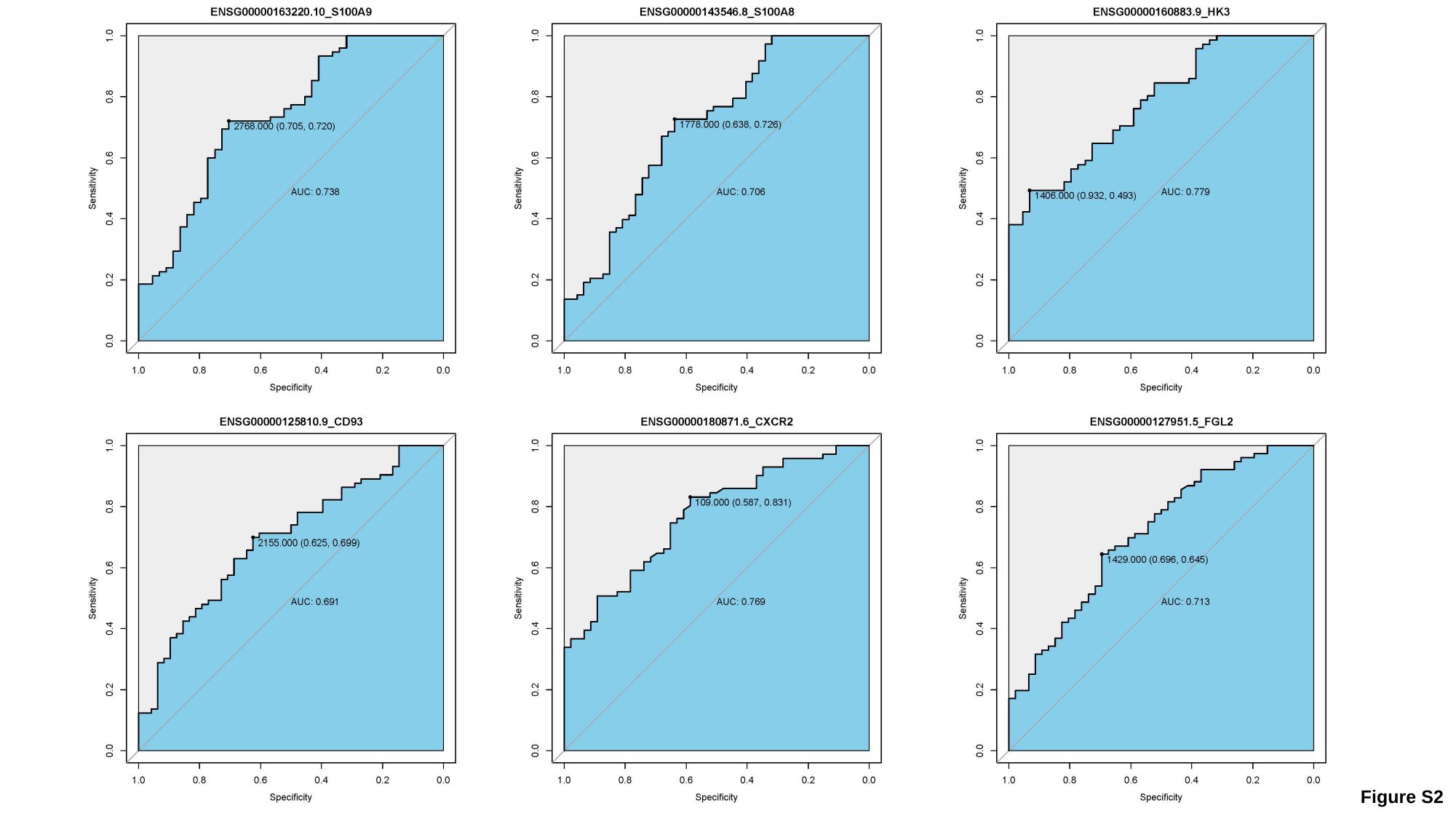

Figure S2

Supplement: Supplementary file 1 — Figure S1. Figure S2. [file JCMM-28-e18552-s003.pptx]
