# Supplementary material for: Identification of two key biomarkers CD93 and FGL2 associated with survival of acute myeloid leukaemia by weighted gene co‐expression network analysis
Source: J Cell Mol Med. 2024 Jul 25;28(14):e18552. doi: 10.1111/jcmm.18552 (PMC11272607; doi:10.1111/jcmm.18552)
Supplement: Supplementary file 2 — Table S1. [file JCMM-28-e18552-s002.docx]

**Table S1 Sample information in this study**

|  | | **AML** | **Normal** |
| --- | --- | --- | --- |
| **Number** | | 100 | 50 |
| **Age (years ± SD)** | | 53.65 ± 16.23 | 54.82 ± 15.82 |
| **Female (%)** | | 52 (52%) | 30 (60%) |
| **Leukemia Morphology** | **M0** | - | 15 |
|  | **M1** | - | 35 |
|  | **M2** | 38 | - |
|  | **M3** | 15 | - |
|  | **M4** | 29 |  |
|  | **M5** | 15 |  |
|  | **M6** | 2 |  |
|  | **M7** | 1 | - |
