# Supplementary material for: Identification of two key biomarkers CD93 and FGL2 associated with survival of acute myeloid leukaemia by weighted gene co‐expression network analysis
Source: J Cell Mol Med. 2024 Jul 25;28(14):e18552. doi: 10.1111/jcmm.18552 (PMC11272607; doi:10.1111/jcmm.18552)
Supplement: Supplementary file 3 — Table S2. [file JCMM-28-e18552-s001.docx]

**Table S2 DNA methylation loci for hub genes**

|  | Probe ID | Chr | Cpg location | Strand | Probe start | Probe end | r | Significance |
| --- | --- | --- | --- | --- | --- | --- | --- | --- |
| S100A9 | cg03165378 | 1 | 153357407 | - | 153357407 | 153357456 | -0.18 | * |
|  | cg23277715 | 1 | 153357593 | - | 153357593 | 153357642 | -0.271 | *** |
|  | cg26937038 | 1 | 153357597 | - | 153357597 | 153357646 | -0.277 | *** |
|  | cg02711163 | 1 | 153357724 | + | 153357676 | 153357725 | -0.283 | *** |
|  | cg05848175 | 1 | 153357782 | - | 153357782 | 153357831 | -0.284 | *** |
|  | cg15160801 | 1 | 153357923 | + | 153357874 | 153357923 | -0.266 | ** |
|  | cg16139316 | 1 | 153358283 | - | 153358283 | 153358332 | -0.291 | *** |
|  | cg20169988 | 1 | 153358300 | + | 153358251 | 153358300 | -0.299 | *** |
|  | cg06355720 | 1 | 153360875 | - | 153360875 | 153360924 | -0.325 | *** |
| HK3 | cg24538331 | 5 | 176884729 | - | 176884729 | 176884778 | 0.205 | * |
|  | cg06485139 | 5 | 176887364 | - | 176887364 | 176887413 | 0.236 | ** |
|  | cg26455624 | 5 | 176896418 | + | 176896369 | 176896418 | -0.217 | ** |
|  | cg17393572 | 5 | 176899364 | + | 176899315 | 176899364 | -0.205 | * |
|  | cg23912072 | 5 | 176899924 | - | 176899924 | 176899973 | -0.2 | * |
|  | cg19791262 | 5 | 176900783 | - | 176900783 | 176900832 | -0.327 | *** |
| CD93 | cg19010566 | 20 | 23082159 | + | 23082110 | 23082159 | -0.544 | *** |
|  | cg05212138 | 20 | 23082482 | + | 23082433 | 23082482 | -0.337 | *** |
|  | cg23815320 | 20 | 23082528 | + | 23082479 | 23082528 | -0.313 | *** |
|  | cg14928764 | 20 | 23083971 | + | 23083922 | 23083971 | -0.382 | *** |
|  | cg22481632 | 20 | 23086308 | - | 23086307 | 23086356 | -0.418 | *** |
|  | cg19138960 | 20 | 23086311 | - | 23086311 | 23086360 | -0.405 | *** |
|  | cg10578007 | 20 | 23086362 | + | 23086313 | 23086362 | -0.38 | *** |
|  | cg10018632 | 20 | 23086397 | - | 23086397 | 23086446 | -0.426 | *** |
|  | cg21023001 | 20 | 23086451 | + | 23086402 | 23086451 | -0.313 | *** |
|  | cg07286123 | 20 | 23086489 | + | 23086440 | 23086489 | -0.304 | *** |
|  | cg20438277 | 20 | 23086519 | + | 23086470 | 23086519 | -0.348 | *** |
|  | cg18267489 | 20 | 23086522 | + | 23086474 | 23086523 | -0.293 | *** |
|  | cg15309910 | 20 | 23087055 | - | 23087054 | 23087103 | -0.636 | *** |
|  | cg12037947 | 20 | 23087115 | + | 23087067 | 23087116 | -0.587 | *** |
|  | cg12873119 | 20 | 23087134 | + | 23087085 | 23087134 | -0.539 | *** |
| CXCR2 | cg10591797 | 2 | 218125876 | - | 218125876 | 218125925 | -0.417 | *** |
|  | cg13739417 | 2 | 218125905 | - | 218125905 | 218125954 | -0.441 | *** |
|  | cg06547715 | 2 | 218126253 | + | 218126204 | 218126253 | -0.478 | *** |
| FGL2 | cg12552626 | 7 | 77198143 | + | 77198094 | 77198143 | -0.201 | * |
|  | cg14752838 | 7 | 77199569 | - | 77199568 | 77199617 | -0.237 | ** |
|  | cg01310473 | 7 | 77199851 | + | 77199802 | 77199851 | -0.253 | ** |
|  | cg23708624 | 7 | 77200528 | + | 77200479 | 77200528 | -0.427 | *** |
|  | cg08241295 | 7 | 77200662 | + | 77200613 | 77200662 | -0.281 | *** |
